# Carbapenem-Resistant Enterobacterales (CRE) in Europe: Surveillance Challenges, Emerging Threats and Public Health Preparedness

**DOI:** 10.3390/antibiotics15080790

**Published:** 2026-08-15

**Authors:** Katarzyna Kuszewska, Karolina Klesiewicz, Paulina Leśniak, Agnieszka Chmielarczyk

**Affiliations:** 1 Diagnostic Laboratory, St. Alexander Hospital, 25-316 Kielce, Poland; kuszewska4@gmail.com; 2 Department of Pharmaceutical Microbiology, Faculty of Pharmacy, Jagiellonian University Medical College, 30-688 Krakow, Poland; paulina.mrowiec@uj.edu.pl; 3 Department of Microbiology, Faculty of Medicine, Jagiellonian University Medical College, 31-121 Krakow, Poland

**Keywords:** carbapenem-resistant Enterobacterales, carbapenemase-producing Enterobacterales, antimicrobial resistance, epidemiological surveillance, genomic surveillance, infection prevention and control, healthcare-associated infections, hypervirulent *Klebsiella pneumoniae*, Europe, public health preparedness

## Abstract

Carbapenem-resistant Enterobacterales (CRE) represent one of the most important antimicrobial resistance threats in Europe, particularly in healthcare settings. Their increasing epidemiological significance is driven by the dissemination of carbapenem-resistant *Klebsiella pneumoniae*, the emergence of high-risk clones, horizontal transfer of mobile genetic elements, and the convergence of antimicrobial resistance and hypervirulence. Despite the growing epidemiological importance of CRE, surveillance and preparedness remain heterogeneous across European countries, creating a need for a continent-wide assessment of existing surveillance systems, implementation gaps, and emerging challenges. This narrative review summarizes the current epidemiology of CRE in Europe and critically discusses the role of European and global surveillance systems in monitoring their spread. Particular attention is given to the European Antimicrobial Resistance Surveillance Network (EARS-Net), European Antimicrobial Resistance Genes Surveillance Network (EURGen-Net), Healthcare-Associated Infections Surveillance Network (HAI-Net), European Surveillance of Antimicrobial Consumption Network (ESAC-Net), Point Prevalence Survey (PPS), EpiPulse/TESSy, the Central Asian and European Surveillance of Antimicrobial Resistance (CAESAR) programme, and the World Health Organization Global Antimicrobial Resistance and Use Surveillance System (WHO GLASS). Although Europe has well-established surveillance infrastructures, substantial heterogeneity persists among countries with respect to microbiological and genomic diagnostic capacity, reporting practices, infection prevention and control (IPC), antimicrobial stewardship, healthcare infrastructure, and implementation of national policies. These differences limit data comparability and may delay the detection of colonization, outbreaks, and cross-border transmission. The available evidence indicates that effective CRE control requires integrated surveillance combining microbiological, genomic, epidemiological, and antimicrobial consumption data within a coordinated One Health framework. Further expansion of genomic surveillance, harmonization of surveillance indicators, strengthening of healthcare system preparedness, and wider implementation of antimicrobial stewardship programmes are identified as priority areas for improve CRE prevention and control across Europe. By integrating evidence across surveillance, diagnostics, infection prevention and control, and antimicrobial stewardship, this review highlights key gaps in current European approaches and provides a framework for prioritizing future surveillance and preparedness efforts.

## 1. Introduction

The increasing prevalence of multidrug-resistant bacterial pathogens poses a substantial challenge to effective infection management and healthcare delivery worldwide [1,2,3]. Since their introduction, antibiotics have served as a cornerstone of modern medicine, significantly reducing morbidity and mortality associated with bacterial infections. Their widespread use in human medicine, veterinary medicine, and agriculture has contributed to the creation of significant selective pressure. This has accelerated the emergence of new resistance mechanisms as well as the activation and spread of existing ones [1,2,3,4,5]. Despite growing awareness of the consequences of the inappropriate and excessive use of antibiotics, this issue remains a top public health priority [3,6].

Carbapenems have long been considered the cornerstone of treatment for severe infections caused by multidrug-resistant Enterobacterales, especially strains producing extended-spectrum β-lactamases (ESβLs) and AmpC β-lactamases, which compromise the activity of many other β-lactam antibiotics [1,7]. They are also used in the treatment of severe infections caused by multidrug-resistant Gram-negative bacilli, such as *Pseudomonas aeruginosa* and *Acinetobacter baumannii* [8]. As a result, they remain among the most important antibiotics used in hospital settings [8,9]. In clinical practice, diagnostic uncertainty and the urgency of initiating treatment frequently result in the overuse of empirical broad-spectrum antibiotic therapy, further accelerating the emergence and dissemination of antimicrobial resistance [10,11].

As a consequence, Enterobacterales have acquired various mechanisms of resistance to carbapenems, leading to the emergence of carbapenem-resistant Enterobacterales (CRE). Among these, the production of carbapenemases represents the most clinically significant mechanism, giving rise to carbapenemase-producing Enterobacterales (CPE). Carbapenemases are β-lactamases capable of hydrolyzing a broad range of β-lactam antibiotics, including carbapenems, thereby compromising the effectiveness of these last-resort agents [7]. According to Ambler’s classification, there are three main classes of carbapenemases: class A serine β-lactamases (including KPC, GES, SME, IMI, NMC-A, FRI), class B metallo-β-lactamases (including NDM, VIM, IMP, SPM, GIM), and class D oxacillinases (including OXA-23, OXA-24/40, OXA-48-like, OXA-58, OXA-143, OXA-181, OXA-232, OXA-235, OXA-244). They differ from one another in terms of substrate spectrum and epidemiological significance [12,13,14,15]. The mechanisms described often co-occur with factors contributing to resistance to other classes of antibiotics. This leads to the selection of bacterial strains with significantly reduced susceptibility (MDR—multidrug-resistant; XDR—Extensively Drug-Resistant) or complete resistance to available drugs (PDR—Pan-Drug-Resistant) [16,17].

This phenomenon represents one of the most serious challenges facing modern medicine and public health. In its document “Bacterial Priority Pathogens List” the World Health Organization (WHO) classifies microorganisms into three priority levels: medium, high, and critical [18]. The group of highest clinical significance includes, among others, carbapenem-resistant Enterobacterales (CRE), ESβL-producing Enterobacterales, and *A. baumannii* [18]. All of these bacteria are closely linked to the use of carbapenems. This underscores the critical importance of measures aimed at limiting and slowing the spread of carbapenemases [13,18,19].

Despite intensive research and development efforts, the global development of new antibiotics remains limited in terms of innovation. Access to existing antimicrobial therapies remains uneven and insufficient [20,21]. Economic analyses show that infections caused by drug-resistant pathogens generate annual global healthcare costs exceeding $100 billion [22]. These figures underscore the fact that the world is facing a phenomenon that is increasingly being referred to as the “Faceless pandemic” [3]. Epidemiological projections indicate that by 2050, antibiotic resistance could be responsible for more than 10 million deaths per year [23].

In an era of intense international human mobility, the links between specific resistance mechanisms and particular regions or countries are subject to very dynamic changes. This underscores the need for systematic surveillance at the local and national levels [24,25]. The first reports of carbapenemases were published in the 1990s in Japan (the first IMP-1-type MBL in *Serratia marcescens*) and in the United States (the first KPC-1 in *K. pneumoniae*) [26,27]. Current data, however, already indicate the global spread of carbapenem-resistant Enterobacterales across all continents [5]. Their apparent absence in some regions should be interpreted with caution, as it often reflects limited diagnostic capabilities and underdeveloped surveillance systems rather than an actual absence in epidemiological terms [28].

Europe represents a particularly complex epidemiological landscape in the context of CRE. Despite relatively high levels of healthcare and social security, the significant impact of social determinants of health continues to be observed. These include, among other things, income and migration inequalities, as well as disparities in access to medical care [29,30,31]. In addition, there are significant differences among European countries regarding infection control strategies, rational antibiotic use, and the diagnostic capabilities of microbiology laboratories [32,33,34,35]. Despite the existence of integrated surveillance systems, coordinated primarily by the European Centre for Disease Prevention and Control (ECDC), there are still significant gaps in the detection and monitoring of transmission [36]. The problem of CRE in Europe is not limited solely to the rise in antibiotic resistance, but also encompasses challenges related to epidemiological surveillance, the effective implementation of control measures, and the early detection of transmission sources in healthcare settings. In this context, the aim of this review was to summarize the evolving epidemiology of CRE in Europe, with a particular focus on challenges related to surveillance systems, public health preparedness, and emerging threats that could influence the further spread of multidrug resistance.

## 2. Epidemiology of CRE in Europe: Surveillance Data

The growing number of infections caused by carbapenem-resistant Enterobacterales places a significant burden on European healthcare systems. This directly affects patient mortality, length of hospital stays, treatment costs, and the limited range of available treatment options [35,37]. In this context, effective epidemiological surveillance is particularly important, as it enables the monitoring of trends and the early detection of changes in the prevalence of CRE in Europe [35,38].

### 2.1. Phenotypic and Genomic Surveillance

Data from the European Antimicrobial Resistance Surveillance Network (EARS-Net) constitute one of the most important sources of epidemiological information on antimicrobial resistance in Europe (covering European Union member states as well as Norway, Iceland, and Liechtenstein-EU/EEA). This system covers only invasive infections (blood and cerebrospinal fluid), and in analyses concerning CRE, it focuses primarily on *E. coli* and *K. pneumoniae* [36]. However, it does not routinely cover other forms of infection or colonization, nor does it include other species belonging to the order Enterobacterales [36,39]. Nevertheless, the 2024 EARS-Net report indicates that the incidence of invasive infections caused by carbapenem-resistant *K. pneumoniae* in Europe has increased by more than 60% compared to 2019 [40]. Between 2019 and 2023, particularly pronounced increases in the estimated incidence were observed in Belgium, Bulgaria, Croatia, Cyprus, the Czech Republic, Germany, Hungary, Latvia, Poland, Romania, Slovakia, and Slovenia. Declining trends were observed only in Finland, Ireland and Malta [35,41]. Furthermore, the data presented are consistent with the 2025 WHO GLASS report, which outlines global trends in antibiotic resistance and also indicates a worldwide upward trend in carbapenem-resistant *K. pneumoniae* [42]. In the European Union and European Economic Area (EU/EEA), carbapenem resistance in *E. coli* remains low (<1% of invasive isolates), whereas globally, its prevalence varies by region [40,42]. In contrast, higher rates have been reported in several Asian regions, particularly in China, where they represent a growing clinical concern [43,44]. Importantly, the GLASS global report, with regard to CRE, includes data not only from bloodstream infections but also from urinary tract infections [42]. However, the EARS-Net and GLASS reports do not routinely provide information on the predominant types of carbapenemases. This is a significant limitation, as the mechanism of resistance is of critical clinical importance and influences the selection of optimal antibiotic therapy, including new drugs active against carbapenemase-producing Enterobacterales [45,46,47].

The European Antimicrobial Resistance Genes Surveillance Network (EURGen-Net), on the other hand, is a surveillance network also coordinated by the ECDC, which serves a complementary role by providing genetic analyses of resistance mechanisms, including carbapenem-degrading enzymes in Enterobacterales. However, EURGen-Net is not a routine system of continuous population surveillance like EARS-Net. It relies primarily on targeted analyses covering selected epidemiological events and research projects, and its geographical coverage and participating countries are not identical to those of EARS-Net [48]. For example, 13 European countries participated in a study of an outbreak involving the transmission of *K. pneumoniae* producing both NDM-1 and OXA-48 carbapenemases between 2014 and 2018. The results indicated a likely widespread distribution of the analyzed clones, potentially extending beyond Europe. It should be emphasized, however, that the limited availability of whole-genome sequencing (WGS) data during this period may have affected the full assessment of the scale of transmission [49]. Subsequent analyses confirmed the ongoing cross-border transmission of CRE, involving various species and resistance mechanisms. A study of *E. coli* producing NDM-5 demonstrated the dominance of high-risk clones and their increasing prevalence. The data were derived from 13 European countries based on strains isolated between 2012 and 2022 [50]. In contrast, a more recent study demonstrated the widespread circulation of a plasmid carrying both the OXA-48 gene and virulence factors among *K. pneumoniae* isolates in many European countries between 2019 and 2024, indicating the significant role of mobile genetic elements in the transmission of resistance [51].

### 2.2. Healthcare-Associated Infections and Antimicrobial Consumption Surveillance

Another surveillance tool developed by the ECDC is the Healthcare-Associated Infections Surveillance Network (HAI-Net). Its main objective is to monitor healthcare-associated infections in EU/EEA countries [52]. Unlike EARS-Net, this system includes data on healthcare-associated infections (HAIs), which are infections that develop during or after the provision of medical care and were not present at the time of the patient’s admission [52,53]. Although HAI-Net largely involves the same EU/EEA countries as EARS-Net, its surveillance scope, participating healthcare facilities, and data collection methodology differ, limiting the direct comparability of data between the two networks. The collected data allow for an assessment of the scale of the problem and the identification of risk factors in various types of facilities. Additionally, it supports the training of hospital infection control teams. Although HAI-Net provides important epidemiological information on the prevalence of multidrug-resistant pathogens, including CRE, it does not include detailed molecular characterization of resistance mechanisms. Furthermore, it does not serve as a direct source of dedicated epidemiological reports, unlike EARS-Net [52].

The European Surveillance of Antimicrobial Consumption Network (ESAC-Net) complements systems that monitor antimicrobial resistance by providing data on antibiotic consumption in EU/EEA countries [54]. To this end, the network primarily uses the international Defined Daily Dose (DDD) indicator, developed by the WHO, which serves as the basic unit for assessing the consumption of antimicrobial drugs. Antibiotic consumption is most often presented as the number of DDDs per 1000 inhabitants per day in outpatient care or as the number of DDDs per 100 patient-days in hospital settings [54,55]. This allows for comparisons of antibiotic use across European countries and analysis of trends over time. The reports produced support the development of strategies to combat antibiotic resistance at the national and international levels. In the context of CRE epidemiology, these data are significant due to the link between the consumption of antibiotics such as carbapenems and the selection of resistant strains, while there is a marked variation in consumption levels across Europe. It should be emphasized, however, that ESAC-Net does not provide microbiological data or information on resistance mechanisms, which limits the direct assessment of associations between antimicrobial consumption and resistance patterns. Nevertheless, it remains an important tool supporting antibiotic policy [54].

The HAI-Net and ESAC-Net networks are collaborating on the European Point Prevalence Survey (PPS), which enables the simultaneous assessment of the prevalence of healthcare-associated infections (HAIs) and antibiotic use in hospitals [56]. The PPS is a standardized cross-sectional survey conducted on a specific date or over a short period of time in healthcare facilities. This survey is an important epidemiological tool because it allows for a comprehensive analysis of the relationship between the incidence of hospital-acquired infections and antibiotic prescribing practices [56,57]. By integrating data on HAIs and antimicrobial consumption, it is possible to identify areas of increased risk for the emergence of multidrug-resistant microorganisms, including carbapenem-resistant Enterobacterales. The latest PPS analysis conducted in 2022–2023 indicated that CRE remain one of the most serious healthcare-associated epidemiological problems in Europe. A particularly high risk of these microorganisms was observed in intensive care units and among long-term hospitalized patients. The report highlighted significant differences among EU/EEA countries in terms of the prevalence of carbapenem-resistant strains. The highest epidemiological burden was mainly observed in Southern and Southeastern European countries [56]. Conversely, the Netherlands, together with several Nordic countries, has consistently maintained a low prevalence of carbapenem-resistant Enterobacterales, reflecting the effectiveness of comprehensive surveillance, prudent antimicrobial use, and stringent infection prevention and control measures [58,59]. Among pathogens, particular significance was attributed to carbapenemase-producing Enterobacterales (especially *K. pneumoniae*), which are a major etiological factor in severe hospital-acquired infections. Analysis of PPS data also indicated a correlation between high consumption of broad-spectrum antibiotics and a higher prevalence of multidrug-resistant microorganisms. The importance of rational antibiotic therapy, effective infection control procedures, and antimicrobial stewardship measures in reducing the transmission of CRE in European healthcare facilities was thus emphasized [56].

### 2.3. Data Integration and Supporting European Surveillance Frameworks

A key element of the standardization and integration of European data is the TESSy/EpiPulse platform, which has been in operation since 2018. It enables the routine collection of information on population coverage, national representativeness, and diagnostic intensity. A common reporting infrastructure for EARS-Net, EURGen-Net, HAI-Net, and ESAC-Net allows for a comprehensive assessment of the epidemiological situation in EU/EEA countries. Furthermore, it enables the analysis of trends in the spread of multidrug-resistant microorganisms, including CRE, and supports the development of strategies to reduce antibiotic resistance at the European and national levels [38]. The table below summarizes the key features of the European surveillance networks coordinated by the ECDC [Table 1].

European efforts to combat antibiotic resistance rely not only on epidemiological surveillance systems, but also on consistent diagnostic and clinical standards. Two organizations play a key role in this area: the European Committee on Antimicrobial Susceptibility Testing (EUCAST) and the European Society of Clinical Microbiology and Infectious Diseases (ESCMID) [61,62]. Although they do not directly participate in the collection of epidemiological data, they provide a methodological and practical foundation for microbiology laboratories and clinicians across Europe [63,64]. EUCAST is responsible for developing and updating European standards for antimicrobial susceptibility testing. This standardization is essential for the comparability of data across countries and for the accurate identification of CRE strains. Importantly, EUCAST guidelines are updated regularly, usually at least once a year, which requires microbiology laboratories to continuously monitor these changes and adapt their diagnostic procedures [62,64,65]. ESCMID, on the other hand, serves as an expert organization that develops clinical guidelines for the diagnosis, treatment, and control of infections. The organization also supports the development of professional competencies through training, courses, and educational programs. This is particularly important given the growing complexity of microbiological diagnostics and the treatment of infections caused by CRE [61,63]. Both organizations are an important part of the infrastructure supporting both the development and practical implementation of measures to limit the spread of multidrug-resistant bacteria in Europe.

It is also worth noting that data from some countries in Eastern Europe and the Balkans (non-EU/EEA), which are not fully represented in the EARS-Net network, are supplemented by WHO initiatives such as the Central Asian and European Surveillance of Antimicrobial Resistance (CAESAR) [66]. However, in recent years, this data has increasingly been incorporated into GLASS’s global analyses rather than published as separate, regular reports [42,66]. The United Kingdom, on the other hand, following its withdrawal from the European Union, reports data on antimicrobial resistance through national channels directly to the WHO’s GLASS system and currently participates in the CAESAR program while remaining outside the ECDC’s surveillance systems [42,67]. In the case of standards developed by EUCAST and ESCMID, several non-EU/EEA countries, such as Switzerland, have officially adopted EUCAST guidelines. In contrast, some countries, including Serbia, have historically used the U.S. CLSI (Clinical and Laboratory Standards Institute) standards in parallel with, or during the transition to, EUCAST [66,68].

**Figure 1:**
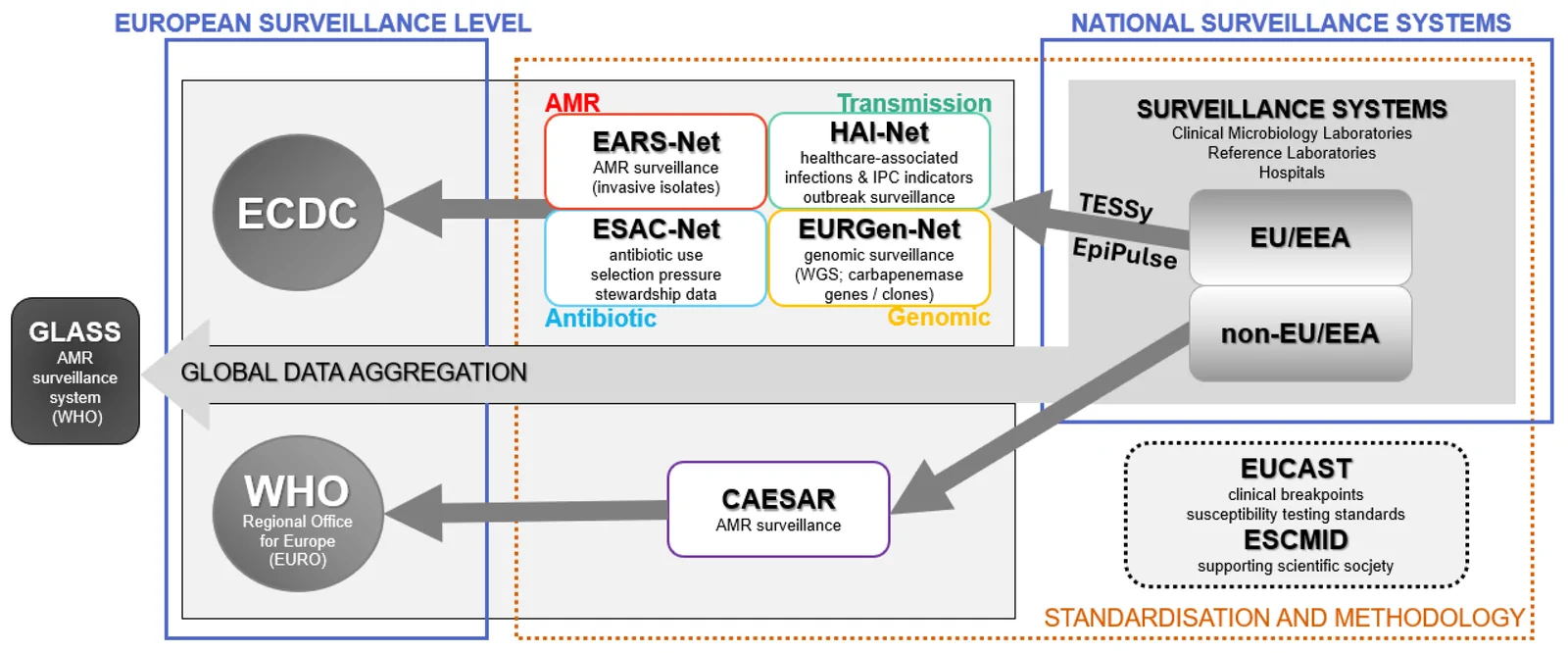
Overview of the European antimicrobial resistance surveillance framework relevant to CRE. National surveillance systems, including clinical microbiology laboratories, reference laboratories and hospitals, provide data to European surveillance networks coordinated by the European Centre for Disease Prevention and Control (ECDC) for EU/EEA countries and by the WHO Regional Office for Europe for non-EU/EEA countries through CAESAR. The European surveillance framework integrates antimicrobial resistance surveillance (EARS-Net), genomic surveillance (EURGen-Net), healthcare-associated infection surveillance (HAI-Net), and antimicrobial consumption surveillance (ESAC-Net). Standardisation of antimicrobial susceptibility testing and interpretation is provided by EUCAST. The dashed line indicates that EUCAST supports methodological standardisation but is not directly involved in data collection or reporting. At the global level, surveillance data contribute to the WHO Global Antimicrobial Resistance and Use Surveillance System (GLASS) for international monitoring and comparison. Source: Author’s own elaboration based on ECDC and global antimicrobial resistance surveillance frameworks [36,42,48,52,54,62,66,69].

To facilitate understanding of the interrelationships among the networks and organizations described above, a simplified diagram illustrating the flow of European CRE surveillance data has been prepared for this review [Figure 1].

## 3. Heterogeneity of Surveillance and Antimicrobial Stewardship Across Europe

### 3.1. Structural and Cross-Country Differences in CRE Control

Despite the existence of coordinated systems in Europe, there are significant differences between countries in terms of strategies to combat AMR (antimicrobial resistance) [60,70]. Socioeconomic factors, public health policy, and guidelines established at the national level are of key importance here [71]. Supervisory, organizational (including infrastructure-related), and educational measures are also a very important aspect [72,73,74]. These differences also reflect varying degrees of implementation of the National Action Plans (NAPs) against AMR, which in many countries remain incomplete or underfunded [75]. Furthermore, one should not overlook the varying degrees of effective enforcement of existing procedures and guidelines regarding infection prevention and control (IPC) [72,73,74]. There are also significant differences in access to diagnostic services and standardized reporting practices. This directly affects the quality and comparability of surveillance data across Europe [70]. Furthermore, in light of the factors mentioned above, the significant differences in antibiotic policies and the extent of overuse and misuse of antimicrobial drugs appear to be particularly important [70,76].

### 3.2. Infection Prevention and Control and Healthcare System Capacity

Infection prevention and control (IPC) measures are among the key strategies for limiting the spread of CRE in healthcare settings [73,74,77]. The particular significance of these procedures stems from the fact that CRE can persist in patients’ gastrointestinal tracts for long periods of time. As a result, these strains may remain unidentified, which facilitates asymptomatic transmission within hospital wards [78,79,80,81]. The level of IPC implementation in Europe varies from country to country, but often also varies among healthcare facilities [82]. These differences are reflected in the epidemiology of CRE across Europe, with endemic transmission reported in several Southern and South-Eastern European countries, whereas sporadic cases and limited outbreaks predominate in Northern Europe where robust IPC measures and coordinated surveillance systems are well established [35,56,58]. Long-term care facilities may also serve as a significant reservoir for CRE colonization and a site of ongoing transmission among patients who have been hospitalized multiple times [83]. In addition, there are still limitations in the availability of microbiological diagnostics, including molecular diagnostics, which are essential for properly assessing the sources and extent of CRE spread [82,84]. The inability to perform timely molecular diagnostic tests (such as PCR) and genomic analyses (such as WGS) leads to delayed detection of CRE [84,85,86]. This is often due to a lack of adequate funding for such activities [35]. In addition, a significant constraint in controlling the spread of CRE is the lack of infrastructure necessary to isolate or cohort patients [87]. Combating AMR requires qualified personnel responsible for epidemiological surveillance at a given facility. Excessive workloads or staff shortages can make it difficult to follow proper procedures [56]. Furthermore, the effective functioning of hospital infection control teams depends largely on national legal regulations. However, not all countries have statutory provisions in place. In such cases, IPC activities are based on existing guidelines, recommendations, or accreditation standards, which can lead to greater variability in the implementation of procedures [82,88]. For example, countries such as the Netherlands, Norway and Sweden have maintained low CRE endemicity through standardized IPC procedures, active screening programmes and coordinated surveillance systems, whereas endemic CRE transmission continues to pose a major challenge in countries such as Greece, Italy and Romania [35,58,59,89]. In some Northern and Western European countries, such as the Netherlands and the Scandinavian countries, the effectiveness of IPC is based not only on legal regulations but also on a high level of standardization of procedures, a strong emphasis on patient safety, and enhanced epidemiological surveillance [58,82].

### 3.3. Antimicrobial Stewardship and Antibiotic Consumption

Monitoring of antibiotic consumption in the EU/EEA region for 2024 shows that the highest rates are observed in Southern European countries and parts of Eastern Europe, while lower consumption levels are characteristic of Northern European countries [54,76]. The biggest problem regarding the sustainable use of antimicrobial medicines is in Greece (29.9 DDD per 1000 inhabitants per day), followed by France (26.5), Romania (25.2), and Spain (24.2). Equally high values are recorded in Malta (23.9), Bulgaria (23.4), Ireland (23.0), and Poland (22.7). Countries with the lowest levels of antibiotic consumption, such as Norway (16.1), Latvia (15.5), Slovenia (14.4), Finland (13.5), and Austria (11.8), are also characterized by a higher degree of rationalization of antibiotic therapy. The data collected by ESAC-Net thus reflect the scale of the problem and allow for the identification of countries requiring intensified efforts to improve antibiotic policy [54,76]. The WHO has developed the AWaRe classification, which divides antibiotics into Access, Watch, and Reserve groups based on the risk of selecting resistant strains. Access antibiotics are preferred for treating the most common infections due to their lower impact on the development of drug resistance, whereas increased use of drugs from the Watch and Reserve groups is considered a factor contributing to the rise in AMR. While the WHO has recommended that Access antibiotics account for at least 60% of total antibiotic consumption [90], a more ambitious target has been established for the European Union. The Council Recommendation of 13 June 2023 on stepping up EU actions to combat antimicrobial resistance in a One Health approach (2023/C 220/01) sets a target for at least 65% of the total consumption of antibiotics in humans in the EU to consist of Access-group antibiotics by 2030 [91]. Current ESAC-Net data demonstrate substantial variation between European countries in the relative consumption of Access-group antibiotics and in the use of broad-spectrum antimicrobial agents [54]. These antibiotics are considered major drivers of CRE emergence and spread because they exert substantial selective pressure on multidrug-resistant Enterobacterales [54,90,92].

**Figure 2:**
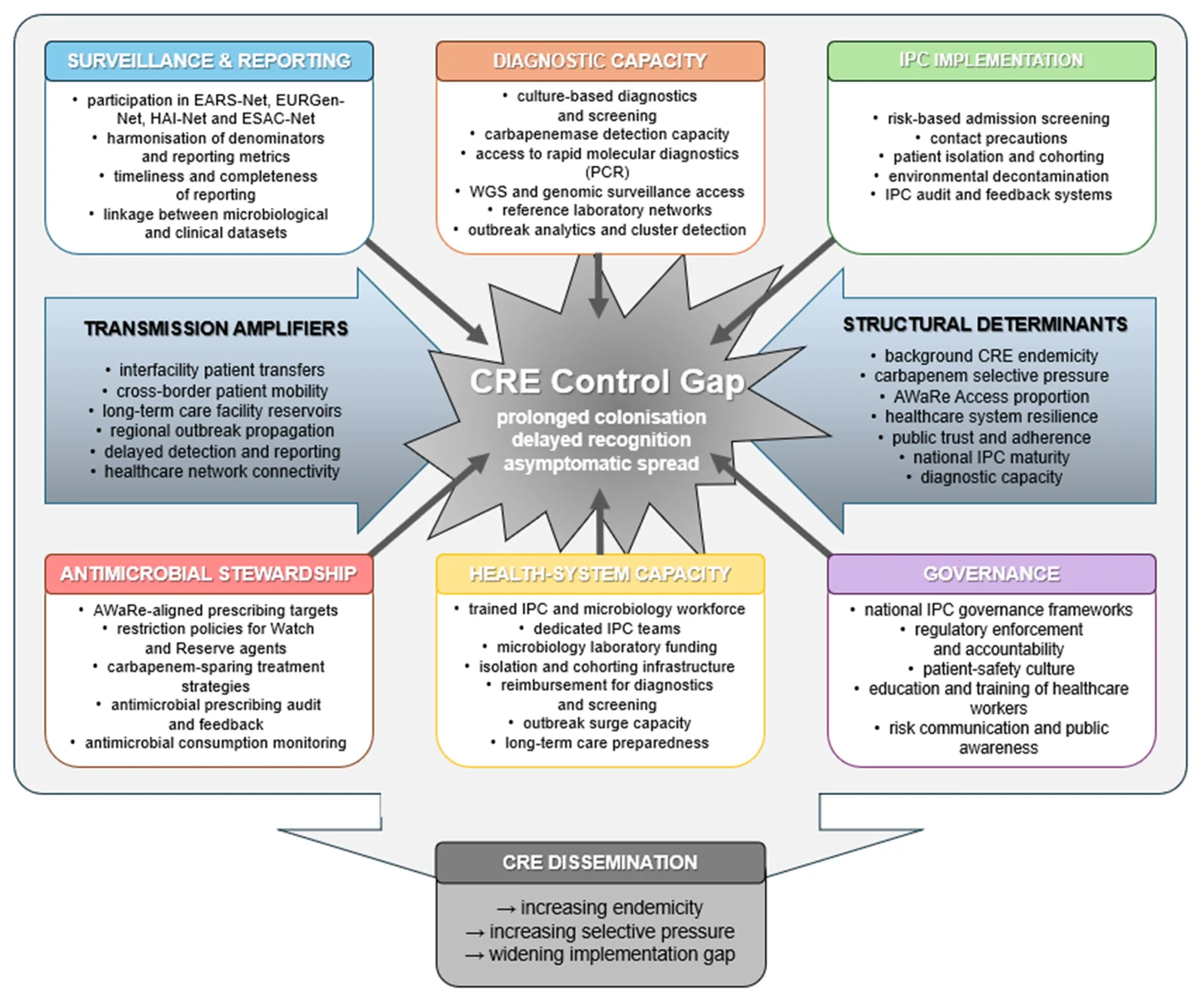
Conceptual framework illustrating the heterogeneity of surveillance, diagnostic capacity, infection prevention and control (IPC), antimicrobial stewardship, governance and health-system capacity across Europe, contributing to gaps in the control of carbapenem-resistant Enterobacterales (CRE). Structural determinants and transmission amplifiers interact with uneven implementation of surveillance, diagnostics and IPC measures, facilitating prolonged colonisation, delayed recognition and asymptomatic spread of CRE within healthcare networks. The resulting dissemination of CRE may further increase endemicity, antimicrobial selective pressure and implementation gaps, creating a self-reinforcing cycle of antimicrobial resistance transmission. Source: Author’s own conceptual framework developed on the basis of ECDC and WHO recommendations, European AMR surveillance systems, and published literature on CRE epidemiology, infection prevention and control (IPC), and antimicrobial stewardship [32,35,36,48,52,54,71,77,90,93].

The observed heterogeneity among European countries indicates that effective control of CRE infections depends not only on the functioning of surveillance systems. The integration of microbiological and genomic diagnostics, IPC measures, and antibiotic policies at the national level is also of key importance. Human and infrastructure resources, the quality of management, and the effectiveness of implementing existing procedures also play a significant role. A lack of coordination among these factors can lead to gaps in CRE control, contributing to delayed detection of colonization and the further transmission of resistant strains within the healthcare system. This is particularly significant in the context of cross-border patient mobility and the operation of interconnected healthcare networks. Local shortcomings in surveillance or diagnostics may facilitate the unnoticed spread of resistant strains at the regional and international levels. This process may be further exacerbated by persistent selective pressure associated with antibiotic use [Figure 2].

## 4. Emerging Threats in CRE

### 4.1. High-Risk and Hypervirulent Klebsiella pneumoniae Lineages

The latest report published in 2025 by the ECDC (which draws its data and conclusions in part from the EARS-Net, EURGen-Net, HAI-Net, as well as other data sources and scientific literature) notes that there has been an increase in the incidence of bloodstream infections caused by carbapenem-resistant *K. pneumoniae* in EU/EEA countries, associated with ongoing transmission in the hospital setting. The increasing spread of hypervirulent *K. pneumoniae* lineages, particularly hvKp ST23-K1, is therefore causing significant epidemiological concern in Europe [35,40,94]. Unlike classic strains of *K. pneumoniae*, hvKp variants are capable of causing severe infections even in previously healthy individuals, often with spread to distant organs [94]. Siderophores involved in iron acquisition, particularly aerobactin, are considered particularly important in the epidemiology of hvKp [51]. The epidemiology of *K. pneumoniae* in Europe is also shaped by the uneven distribution of internationally disseminated high-risk lineages. Major clones associated with carbapenem resistance include ST258/512, ST307, ST147, ST101, and ST11, although their relative prevalence differs substantially between European countries and regions [95,96]. This geographical heterogeneity is epidemiologically relevant because the acquisition of carbapenemase genes and virulence determinants occurs within specific clonal backgrounds rather than uniformly across the *K. pneumoniae* population [95,97,98]. Consequently, surveillance based solely on resistance phenotypes or carbapenemase types may not fully capture the emergence and cross-border spread of high-risk clones. Another cause for concern is the convergence of virulence factors and antimicrobial resistance, which may lead to the emergence of strains with limited therapeutic options. With the spread of carbapenem-resistant hvKp in healthcare settings, particularly among high-risk patient populations, a further increase in morbidity and rising mortality rates is to be expected [94].

### 4.2. Carbapenem Resistance Beyond Klebsiella pneumoniae and Escherichia coli

A very important issue that remains insufficiently addressed in European epidemiological surveillance systems is the fact that, in addition to *K. pneumoniae* and *E. coli*, other species belonging to the order Enterobacterales may also acquire carbapenem resistance mechanisms [35]. Scientific publications have described the presence of carbapenem-resistant strains of, among others, the *Enterobacter cloacae* complex, *Citrobacter* spp., *Serratia marcescens*, *Morganella morganii*, *Providencia* spp., *K. aerogenes*, *K. oxytoca*, and *Proteus* spp. [35,99,100,101,102]. Importantly, these microorganisms are among the most commonly identified causative agents of infections in humans [103,104,105]. Furthermore, in some of these species (*Proteus* spp., *Morganella* spp., *Providencia* spp., and *Serratia* spp.), a naturally reduced susceptibility to polymyxins, including colistin, which is considered a so-called “last-resort” antibiotic, has also been observed [106,107]. The co-occurrence of carbapenem resistance and the limited efficacy of colistin severely limits treatment options [108,109]. Currently, ECDC surveillance systems focus primarily on selected priority pathogens, which are responsible for the highest number of invasive or severe infections, due in part to organizational and diagnostic limitations. This means that European epidemiological reports may not reflect the full extent of carbapenem resistance among all Enterobacterales [35].

### 4.3. Diagnostic Challenges, Mobile Genetic Elements, and Silent Dissemination

In addition, there are laboratory limitations associated with conventional testing of strains for carbapenemase production (particularly OXA-48-like variants, GES, and certain KPCs). This is due to a slight increase in carbapenem MIC (minimum inhibitory concentration) values, the maintenance of phenotypic susceptibility, or the lack of specific tests in routinely used diagnostic methods [110,111,112,113,114,115]. An additional challenge is the varying levels of resistance gene expression, which can affect the phenotypic detectability of strains [116]. It has also been shown that the simultaneous loss of porins, combined with low levels of carbapenemase expression, can often lead to false-negative results [117]. In practice, this can lead to the persistence of unidentified transmission reservoirs in healthcare facilities [112,114,115,118,119].

Mobile genetic elements involved in horizontal gene transfer (HGT) are playing an increasingly important role in the epidemiology of CRE [13,35]. Of particular concern is the emergence of so-called mosaic plasmids, which can simultaneously carry carbapenemase genes and hypervirulence determinants [51,120,121]. This phenomenon indicates that the epidemiological threat now stems not only from bacterial strains themselves, but also from mobile genetic elements that enable the rapid spread of resistance and virulence traits within the hospital environment [51,122]. Unlike classical clonal transmission, horizontal plasmid transfer can involve many different species belonging to the Enterobacterales [101,123,124]. This could potentially accelerate the emergence of strains that pose a high epidemic risk and, at the same time, have limited treatment options. Furthermore, the dynamic nature of these processes may hinder effective epidemiological surveillance based solely on monitoring resistance types or phenotypes [35,125].

Current CRE surveillance strategies focus primarily on detecting established infections. This approach does not fully reflect the dynamics of how resistance mechanisms spread within the healthcare setting [126]. Increasing importance is being attributed to so-called “silent dissemination” in which the transmission of multidrug-resistant strains can occur even before clinically apparent infections emerge. This process is associated with asymptomatic colonization of patients with CRE strains and the transfer of resistance genes between species [124,126]. Current guidelines identify patient groups requiring special monitoring, including, among other measures, screening upon hospital admission and the use of contact isolation until microbiological test results are available [73,93,127]. However, the effective implementation of such procedures requires adequate infrastructure, funding for a large number of daily tests, and qualified personnel to oversee the process. In many European healthcare systems, meeting these standards remains a challenge. It is worth remembering, however, that even a single undetected CRE carrier can become a source of further transmission and new outbreaks [35,73,126].

## 5. Discussion

### 5.1. Challenges and Priorities for CRE Surveillance and Control in Europe

The information presented on European surveillance systems and the data derived from them highlights a number of key challenges. The epidemiology of CRE in Europe remains a dynamic phenomenon, with significant geographical and structural variation. Despite the intensive efforts already undertaken to combat AMR, persistent differences between countries and healthcare facilities contribute to the continued colonization of patients and the healthcare environment by multidrug-resistant microorganisms [33,35,70]. The current situation highlights the need to harmonize national regulations and establish internationally consistent systems to oversee the actual implementation of diagnostic and therapeutic standards and IPC procedures [73,82,93]. Of all the identified barriers, the lack of harmonization of surveillance systems and diagnostic and epidemiological practices is the most significant. This prevents data comparability, delays the detection of transmission, and limits the effectiveness of interventions at both the local and international levels [32,35,38,70].

The current CRE surveillance networks in Europe also do not reflect the actual burden on the population. Surveillance limited solely to hospitals does not reflect the full extent of transmission [35]. Wastewater surveillance may provide complementary information on the circulation of carbapenem-resistant bacteria and resistance determinants outside conventional clinical surveillance [128,129,130,131]. At the European level, wastewater-based surveillance of antimicrobial resistance is increasingly moving from a research-oriented approach toward formal implementation. The recast Urban Wastewater Treatment Directive (Directive (EU) 2024/3019, Article 17) introduces requirements for monitoring antimicrobial resistance in urban wastewater from agglomerations of at least 100,000 population equivalents (PE), with implementation obligations being progressively phased in towards 2030 [132]. However, a substantial implementation gap remains across Europe. Methodological guidance regarding surveillance scope, targets, and indicators is still under development, while a 2024 EU-WISH mapping survey found that only 11 of 27 surveyed European countries had an operative wastewater-based surveillance system for AMR [133]. Therefore, the key challenge is no longer solely to expand environmental studies, but to harmonize methodologies and translate the emerging regulatory framework into sustainable and comparable surveillance systems across Europe. In the context of CRE epidemiology, the need to adopt a “One Health” perspective is therefore clearly evident. This concept stems from the observation of close links between human, animal, and environmental health [122,123,124]. Understanding these links is crucial, as measures to limit the transmission of multidrug-resistant bacteria in healthcare facilities are insufficient unless other sources are also addressed. Therefore, the integration of clinical, environmental, and veterinary data must be the next strategic step in the fight against AMR [130,134].

It is becoming increasingly clear that the current epidemiology of CRE goes beyond the capabilities of traditional surveillance, which relies primarily on the detection of invasive infections and phenotypic assessment of drug susceptibility [35,84,85,126]. Phenomena such as long-term colonization of patients, silent dissemination, horizontal transfer of resistance genes, and the spread of mobile genetic elements can lead to the persistence of undetected reservoirs of transmission within the hospital environment [79,80,122,123,124].

However, the interpretation of epidemiological data on CRE in Europe remains limited by the heterogeneity of reporting systems and differences in the availability of molecular diagnostics. An additional challenge is the uneven implementation of genomic surveillance across countries [32,35,85].

Examples from countries with well-established AMR surveillance systems indicate the direction in which European CRE surveillance should develop. The Netherlands provides an example of integrated national monitoring by combining data on antimicrobial consumption, antimicrobial resistance, and genomic surveillance in humans and animals through the Netherlands Map of Antimicrobial Resistance (NethMap) and the Monitoring of Antimicrobial Resistance and Antibiotic Usage in Animals in the Netherlands (MARAN). In addition, whole-genome sequencing is routinely used for surveillance of multidrug-resistant organisms [58,89,135]. Denmark has developed a long-standing One Health surveillance model through Danish Integrated Antimicrobial Resistance Monitoring and Research Programme (DANMAP), which systematically monitors antimicrobial use and resistance in humans, animals and food [136]. Sweden represents another useful benchmark, with low AMR levels supported by coordinated national surveillance, prudent antimicrobial use, robust IPC programmes and strong collaboration between public health and veterinary sectors [137]. Together, these systems demonstrate the benefits of integrating microbiological, epidemiological, antimicrobial consumption and genomic data within a coordinated national One Health surveillance framework [35].

The broader implementation of WGS technology in microbiology laboratories therefore remains crucial [84,85]. In addition to identifying resistance genes, WGS enables the detection of high-risk clones, the analysis of transmission, and more accurate comparisons of international data [84,85,138]. The results of the CCRE Survey further support the importance of this approach [96,138,139]. The CRE25 project is currently underway; it is the third European study on carbapenem-resistant Enterobacterales [69,84]. Nevertheless, genomic surveillance of AMR should become part of the routine monitoring system in Europe [84,85,140].

In practice, effective control of CRE currently requires a shift from reactive detection of established infections to a more integrated and proactive surveillance model. This should include genomic diagnostics, monitoring of colonization, infection prevention and control (IPC) measures, and analysis of transmission between the environment, animals, and humans [35,73,84,85,134].

A key contribution of this review is the integration of multiple dimensions of CRE surveillance that are often considered separately, including phenotypic resistance monitoring, genomic surveillance, healthcare-associated infection surveillance, antimicrobial consumption, infection prevention and control, and emerging One Health approaches. This integrated perspective highlights that a major challenge for CRE surveillance in Europe is not solely the availability of surveillance systems, but also their heterogeneity, variable implementation, and limited integration across countries and surveillance domains [32,35,70,85]. Taken together, these findings indicate that greater harmonization and integration of microbiological, genomic, epidemiological, and antimicrobial consumption data, combined with strengthened infection prevention and antimicrobial stewardship, should be considered key priorities for improving European preparedness for CRE [35,85,134,135,136].

In addition, emphasis should also be placed on collaboration and education among all healthcare professionals—from doctors, nurses, microbiologists, and pharmacists, to cleaning and support staff. Each of these groups plays a vital role in maintaining high standards in the fight against AMR [141,142,143]. Only coordinated efforts, based on mutual respect, can lead to lasting solutions. The lack of interdisciplinary cooperation significantly limits the effectiveness of even the best-designed strategies [73,88,141,142]. This aspect is often underestimated, yet it serves as the true foundation for the effective work of hospital infection control teams [33,88,141].

### 5.2. Recommendations for CRE Prevention in Europe

Based on the evidence reviewed, prevention of CRE in Europe should rely on a coordinated, multi-level approach [35]. Priority actions include harmonization of surveillance definitions and reporting practices across countries, broader integration of genomic surveillance into routine monitoring, improved detection of asymptomatic colonization and transmission, and consistent implementation of infection prevention and control measures [35,70,85]. Antimicrobial stewardship should be strengthened through closer monitoring of antibiotic consumption and more rational use of broad-spectrum agents [35,54]. In addition, environmental and One Health surveillance, including wastewater monitoring, should be integrated with clinical and epidemiological data [132,133,134]. Finally, sustained investment in diagnostic capacity, trained personnel, healthcare infrastructure, and interdisciplinary collaboration is essential to translate surveillance data into timely preventive action [35,77,142].

### 5.3. Limitations

Several limitations of this narrative review should be acknowledged. First, due to its narrative design, the review was not based on a systematic literature search and study-selection protocol; therefore, some relevant evidence may not have been captured. Second, the broad geographical scope encompasses European countries with heterogeneous healthcare systems, surveillance capacities, diagnostic practices, and reporting structures, which limits the possibility of providing equally detailed assessments for individual countries or sub-regions. However, this broad scope was retained intentionally, as identifying such heterogeneity and its implications for the comparability and integration of CRE surveillance is one of the central objectives of this review. Third, differences in surveillance methodologies, case definitions, diagnostic capacity, and reporting practices across countries may limit direct comparisons of epidemiological data. Finally, the epidemiology of CRE and the European surveillance landscape are evolving rapidly, particularly with the expansion of genomic surveillance and emerging regulatory initiatives; consequently, some surveillance structures, policies, and epidemiological patterns may change as new data become available.

## 6. Future Directions

In the coming years, it will be crucial to develop tools that enable a more proactive and integrated approach to CRE surveillance.

### 6.1. Digital and Data-Driven Surveillance

In an era marked by the development of complex IT systems and artificial intelligence (AI), solutions that help identify patients at risk of colonization by multidrug-resistant bacteria (CRE triage) may become increasingly important [144]. Many medical facilities are experiencing significant staff workload, which exceeds available personnel and organizational resources. In such situations, this can lead to oversights [56]. In recent years, models have been developed in the United States, South Korea, Europe, and China, among other places, that analyze data from electronic health records and automatically identify individuals who require screening [144,145,146,147]. These solutions are still in the pilot phase, but their effectiveness suggests that they could become an important component of future strategies for infection control and surveillance of CRE [145,148].

Monitoring antibiotic use at the patient level also remains an important area of focus. Currently, there is significant variation in this regard across Europe [54]. Some countries already have prescription-tracking systems in place, others are in the process of implementing them, and still others do not yet have such capabilities. This significantly hinders efforts to optimize antibiotic therapy [71]. Patient-level monitoring of antibiotic use is widely recommended as a means of improving the quality of care, and efforts should be made to implement it effectively [143]. In the future, solutions that enable more precise monitoring, such as real-time dose-level tracking, may prove particularly valuable [149,150].

### 6.2. Integrated and Emerging Prevention Strategies

Beyond technological innovations, increasing attention is also being paid to integrated implementation strategies that combine surveillance with infection prevention and antimicrobial stewardship. The Horizon 2020 REVERSE project is evaluating the implementation of integrated microbiology and diagnostic stewardship, infection prevention and control, and antimicrobial stewardship programmes across hospitals in several European countries. Such implementation-focused initiatives may generate valuable evidence to inform integrated strategies for the prevention and control of carbapenem-resistant Enterobacterales and other multidrug-resistant pathogens [151].

Increasing attention is also being paid to the role of local hospital wastewater pretreatment facilities. These facilities serve as point sources of high concentrations of multidrug-resistant microorganisms, and conventional municipal wastewater treatment plants are not designed to effectively reduce resistance genes or carbapenemases [140,141]. Projects such as THERESA PCP and pilot studies conducted in Switzerland indicate that preliminary wastewater treatment can reduce CRE emissions into the environment and serve as a valuable complement to clinical monitoring [152,153,154].

In the future, interventions aimed at modulating the gut ecosystem of patients colonized with CRE—including fecal microbiota transplants (FMT) and selected probiotic preparations—may also prove important. These interventions may support the restoration of normal gut microbial composition and limit long-term colonization by carbapenem-resistant Enterobacterales, although their efficacy in this context requires further study [78,155,156].

In the long term, the full implementation and funding of NAPs will also be crucial, as this will enable the harmonization of surveillance systems and a more effective response to CRE-related risks [75].

## 7. Literature Search Strategy

This narrative review summarizes the current evidence regarding carbapenem-resistant Enterobacterales (CRE) in Europe, with a particular focus on epidemiology, surveillance systems, resistance mechanisms, and public health preparedness. A literature search was conducted using the PubMed/MEDLINE, Scopus, Web of Science, and Google Scholar databases, focusing primarily on publications published between 2018 and May 2026. Earlier landmark studies were included where relevant to provide historical context and to describe the evolution of carbapenem resistance mechanisms and European surveillance systems.

The search strategy used combinations of keywords related to carbapenem-resistant Enterobacterales, antimicrobial resistance, carbapenemases, epidemiology, surveillance, antimicrobial stewardship, infection prevention and control, genomic surveillance, antimicrobial consumption, public health preparedness, and novel therapeutic options. Examples of search terms included carbapenem-resistant Enterobacterales, CRE, carbapenemase-producing Enterobacterales, CPE, antibiotic resistance, carbapenemase, EARS-Net, GLASS, CAESAR, antibiotic consumption Europe, WHO Priority Pathogens, hvKp ST23, One Health, and whole-genome sequencing (WGS).

In addition, official reports, guidelines, and technical documents published by the European Centre for Disease Prevention and Control (ECDC), the World Health Organization (WHO), the European Committee on Antimicrobial Susceptibility Testing (EUCAST), the European Society of Clinical Microbiology and Infectious Diseases (ESCMID), and European and international antimicrobial resistance surveillance systems, including EARS-Net, EURGen-Net, HAI-Net, ESAC-Net, and GLASS, were also reviewed. The retrieved literature was critically evaluated and synthesized narratively according to its relevance to the scope and objectives of this review.

## 8. Conclusions

Carbapenem-resistant Enterobacterales remain one of the most significant threats posed by antibiotic resistance in Europe and worldwide. Despite the existence of extensive epidemiological surveillance systems, persistent heterogeneity in diagnosis, reporting, and the capacity to implement infection prevention and control (IPC) measures continues to limit the effectiveness of CRE transmission control across European healthcare systems.

Of particular concern is the growing importance of hypervirulent and carbapenem-resistant strains of *K. pneumoniae*, as well as the potentially underestimated role of other Enterobacterales, which are capable of acquiring and spreading carbapenemases. Increasing importance is also being attributed to mobile genetic elements involved in the spread of resistance mechanisms. These phenomena indicate that future surveillance strategies should go beyond traditional monitoring of phenotypic resistance and include the integration of genomic, epidemiological, and clinical data.

Effectively curbing the further spread of CRE in Europe will require further integration of epidemiological surveillance, the development of genomic diagnostics, intensified IPC measures, and the continued rational use of antibiotics. Other essential elements include sustained institutional support, coordinated policy actions (both national and international), and raising awareness of the issue at all levels of the healthcare system.

## Figures and Tables

**Table 1:** Comparison of ECDC surveillance networks relevant to CRE epidemiology in Europe.

Characteristic	EARS-Net	EURGen-Net	HAI-Net	ESAC-Net
Primary objective	Monitoring AMR trends	Detection of resistance genes and transmission pathways	Surveillance of healthcare-associated infections	Monitoring antimicrobial consumption
Type of data	Phenotypic (susceptibility testing)	Genomic (WGS, resistance genes)	Epidemiological, clinical	Quantitative (DDD, prescriptions)
Population coverage	EU/EEA	Selected countries/studies	EU/EEA	EU/EEA
Clinical scope	IF (blood, cerebrospinal fluid)	Selected isolates (targeted sampling)	HAI	Community and hospital antibiotic use
Includes colonisation data	No	Sometimes (targeted studies)	Limited (depending on survey)	No
Includes non-invasive infections	No	Yes (selected)	Yes	Not applicable
Temporal resolution	Continuous	Episodic/project-based	Continuous + PPS	Continuous
Strengths	High comparability, trend analysis	High-resolution molecular insight	Burden of disease, healthcare context	Links antibiotic use with resistance pressure
Key limitations	Underestimates total burden (only invasive cases); no molecular data	Limited representativeness; not population-based	Limited microbiological detail; variability between surveys	No direct linkage to resistance or outcomes
CRE-specific data	Direct (*K. pneumoniae*, *E. coli*)	Direct (carbapenemases, clones)	Indirect (clinical infections, outbreaks)	No direct CRE data
Role in CRE surveillance	Quantifies burden and trends	Identifies mechanisms and cross-border spread	Explains transmission in healthcare settings	Provides context of selective pressure (antibiotic use)

Abbreviations: CRE—Carbapenem-Resistant Enterobacterales; ECDC—European Centre for Disease Prevention and Control; EARS-Net—European Antimicrobial Resistance Surveillance Network; EURGen-Net—European Genomic Surveillance Network; HAI-Net—Healthcare-Associated Infections Surveillance Network; ESAC-Net—European Surveillance of Antimicrobial Consumption Network; EU/EEA—European Union/European Economic Area; AMR—antimicrobial resistance; WGS—whole genome sequencing; DDD—defined daily dose; IF—invasive infections; HAI—healthcare-associated infections; PPS—point prevalence survey. Source: Author’s own elaboration based on ECDC surveillance frameworks [36,48,52,54,56,60].

## Data Availability

No new data were created or analyzed in this study. Data sharing not applicable to this article.

## Abbreviations

The following abbreviations are used in this manuscript:

AI	artificial intelligence
AMR	antimicrobial resistance
AWaRe	Access, Watch, and Reserve
CAESAR	Central Asian and European Surveillance of Antimicrobial Resistance
CPE	carbapenemase-producing Enterobacterales
CRE	carbapenem-resistant Enterobacterales
DANMAP	Danish Integrated Antimicrobial Resistance Monitoring and Research Programme
DDD	Defined Daily Dose
EARS-Net	European Antimicrobial Resistance Surveillance Network
ECDC	European Centre for Disease Prevention and Control
EpiPulse	European surveillance portal for infectious diseases
ESAC-Net	European Surveillance of Antimicrobial Consumption Network
ESβLs	specially strains producing extended-spectrum β-lactamases
ESCMID	the European Society of Clinical Microbiology and Infectious Diseases
EUCAST	the European Committee on Antimicrobial Susceptibility Testing
EU/EEA	European Union, Norway, Iceland and Liechtenstein
EURGen-Net	European Antimicrobial Resistance Genes Surveillance Network
FMT	fecal microbiota transplants
GLASS	Global Antimicrobial Resistance and Use Surveillance System
HAI-Net	Healthcare-Associated Infections Surveillance Network
HAIs	healthcare-associated infections
HGT	horizontal gene transfer
hvKp	hypervirulent *Klebsiella pneumoniae*
IF	invasive infections
IMP	Imipenemase
IPC	infection prevention and control
KPC	*Klebsiella pneumoniae* carbapenemases
MARAN	Monitoring of Antimicrobial Resistance and Antibiotic Usage in Animals in the Netherlands
MBL	Metallo-β-lactamase
MDR	multidrug-resistant
NAPs	the National Action Plans
NDM	New Delhi metallo-β-lactamase
NethMap	Netherlands Map of Antimicrobial Resistance
OXA	Oxacillinase
PCR	polymerase chain reaction
PDR	Pan-Drug-Resistant
PPS	Point Prevalence Survey
TESSy	The European Surveillance System
WGS	whole-genome sequencing
WHO	World Health Organization
VIM	Verona integron-encoded metallo-β-lactamase
XDR	Extensively Drug-Resistant
